# Impact of Different Treatment Regimens and Timeframes in the Plasmatic Metabolic Profiling of Patients with Lung Adenocarcinoma

**DOI:** 10.3390/metabo13121180

**Published:** 2023-11-30

**Authors:** Daniela Madama, David F. Carrageta, Bárbara Guerra-Carvalho, Maria F. Botelho, Pedro F. Oliveira, Carlos R. Cordeiro, Marco G. Alves, Ana M. Abrantes

**Affiliations:** 1 Clinical Academic Centre of Coimbra (CACC), Department of Pulmonology, Faculty of Medicine, University Hospitals of Coimbra, University of Coimbra, 3004-504 Coimbra, Portugal; 2 Clinical and Experimental Endocrinology, UMIB—Unit for Multidisciplinary Research in Biomedicine, ICBAS—School of Medicine and Biomedical Sciences, University of Porto, 4050-313 Porto, Portugalalvesmarc@gmail.com (M.G.A.); 3 Laboratory for Integrative and Translational Research in Population Health (ITR), University of Porto, 4050-600 Porto, Portugal; 4 LAQV-REQUIMTE, Department of Chemistry, University of Aveiro, 3810-193 Aveiro, Portugal; p.foliveira@ua.pt; 5 Clinical Academic Centre of Coimbra (CACC), Centre for Innovative Biomedicine and Biotechnology (CIBB), Coimbra Institute for Clinical and Biomedical Research (iCBR), Biophysics Institute of Faculty of Medicine of University of Coimbra, Area of Environmental Genetics and Oncobiology (CIMAGO), 3000-548 Coimbra, Portugal

**Keywords:** lung adenocarcinoma, chemotherapy, immunotherapy, metabolomics

## Abstract

In recent years, the treatment of advanced non-small cell lung cancer (NSCLC) has suffered a variety of alterations. Chemotherapy (CTX), immunotherapy (IT) and tyrosine kinase inhibitors (TKI) have shown remarkable results. However, not all patients with NSCLC respond to these drug treatments or receive durable benefits. In this framework, metabolomics has been applied to improve the diagnosis, treatment, and prognosis of lung cancer and particularly lung adenocarcinoma (AdC). In our study, metabolomics was used to analyze plasma samples from 18 patients with AdC treated with CTX or IT via ^1^H-NMR spectroscopy. Relevant clinical information was gathered, and several biochemical parameters were also evaluated throughout the treatments. During the follow-up of patients undergoing CTX or IT, imaging control is recommended in order to assess the effectiveness of the therapy. This evaluation is usually performed every three treatments. Based on this procedure, all the samples were collected before the beginning of the treatment and after three and six treatments. The identified and quantified metabolites in the analyzed plasma samples were the following: isoleucine, valine, alanine, acetate, lactate, glucose, tyrosine, and formate. Multivariate/univariate statistical analyses were performed. Our data are in accordance with previous published results, suggesting that the plasma glucose levels of patients under CTX become higher throughout the course of treatment, which we hypothesize could be related to the tumor response to the therapy. It was also found that alanine levels become lower during treatment with CTX regimens, a fact that could be associated with frailty. NMR spectra of long responders’ profiles also showed similar results. Based on the results of the study, metabolomics can represent a potential option for future studies, in order to facilitate patient selection and the monitoring of therapy efficacy in treated patients with AdC. Further studies are needed to improve the prospective identification of predictive markers, particularly glucose and alanine levels, as well as confer guidance to NSCLC treatment and patient stratification, thus avoiding ineffective therapeutic strategies.

## 1. Introduction

Lung cancer is a significant health issue in contemporary society, as it continues to be the primary cause of cancer-related deaths worldwide [1]. Despite a slight decrease in Western countries, the incidence and mortality rates of lung cancer are on the rise [1]. While smoking remains one of the primary risk factors, contributing to 25% of cases, it’s worth noting that 15% of lung cancers in men and 53% in women are not associated with smoking [1]. It has shown a decrease in incidence and mortality in recent decades, in some countries—such as the United States. However, the incidence is expected to rise further in others—such as China—where there has been a dramatic increase in smoking during recent decades [2].

Early diagnosis of cancer can significantly improve a patient’s chance of survival, with higher five-year survival rates reported when the cancer is detected in its initial stages [3]. Unfortunately, more than 61% of patients are diagnosed at advanced stages (III and IV), when treatment options are limited. In these cases, the five-year survival rate drops considerably, and the five-year survival rate can be as low as 4% [3].

These statistics show the extreme importance of the search for biomarkers that could be used in the early detection of lung cancer. No biomarkers have been clinically utilized in lung cancer diagnosis, but it is an important topic of research.

Depending on the stage of the disease, lung cancer therapy has been based on classical chemotherapy (CTX) regimens for many years, as well as radiotherapy and surgical therapy [4]. Combination CTX regimens produce 1-year survival rates of 30% to 40% and are more efficacious than single agents [5]. The approval of tyrosine kinase inhibitors (TKIs) revolutionized therapeutic guidelines, as they provide a personalized therapy [4]. The relationship between cancer and the immune system has been extensively studied, which has led to the identification of molecular mechanisms used by cancer cells to incorporate certain T-cell receptors [6]. This mechanism is known to prevent the cytotoxic response which protects from the antitumor immune attack [6]. The use of immune checkpoint inhibitors is one of the most significant and important improvements of the last years in cancer treatment [7,8]. Patients with Non-Small cell Lung Cancer (NSCLC) who are eligible for targeted therapies or immunotherapies are now surviving longer; 5-year survival rates range from 15% to 50%, depending on the biomarker [5].

Results obtained with programmed cell death protein-1 (PD-1) and programmed death-ligand 1 (PD-L1) inhibitors have shown remarkable and durable clinical activity in patients with advanced NSCLC, with few adverse reactions [9]. Such impressive responses have led to multiple changes in the therapeutic algorithm of advanced NSCLC, with new first-line treatment options for patients with tumors positive for PD-L1 [5], as well as combinations with CTX and second-line options after initial CTX regimens. In recent years, randomized trials using IT showed positive results compared with standard CT [10,11]. One example is KEYNOTE-024, a phase 3 randomized trial, which compared single-agent pembrolizumab versus platinum-based CTX as first-line therapy for patients with advanced nonsquamous or squamous NSCLC and PD-L1 expression levels of 50% or more but without mutations. At 6 months, the rate of overall survival (OS) was 80.2% with pembrolizumab monotherapy versus 72.4% with CTX [12]. So, IT is considered one of the most important breakthroughs in cancer treatment of the past decade; notably, different studies of PD-1 and PD-L1 inhibitors have reported impressive clinical activity and durable responses in patients with advanced NSCLC. These findings have led to the changing of the current therapeutic algorithm of advanced NSCLC, adding a new standard first-line treatment option for patients with PD-L1-positive tumors [11], combination regimens of CTX and IT, and also as a second-line option after an initial CTX regimen. In the KEYNOTE-189 trial, the estimated rate of OS at one year was 69.2% (95% CI, 64.1–73.8%) in patients receiving pembrolizumab plus CTX versus 49.4% (95% CI, 42.1–56.2%) for CTX alone [13].

Although responses are surprising and durable, they are observed only in a subset of unselected patients [7]. It is becoming increasingly important to identify predictive and prognostic biomarkers to guide patient selection. A good biomarker must have both analytical validity, meaning it is reliable and reproducible, as well as clinical utility [14]. However, identifying reliable methods for predicting Immune Checkpoint Inhibitors response has been challenging, and there are varying results from recent efforts in this area [15,16].

The metabolic reprogramming of tumor cells to support continuous growth and proliferation has been recognized as an emergent hallmark in cancer development [17]. There is cumulative evidence that many solid tumors present altered metabolic pathways, such as enhanced glucose uptake and glycolytic activity, increased de novo biosynthesis of nucleotides, increased glutaminolysis, or a shift in citrate metabolism from oxidation to lipogenesis [18,19]. Over the last few years, interest in metabolomics in the study of lung cancer has increased [3]. One of the most important advantages of this technique is that it allows using different types of samples, such as cultured cells, tissues, and biofluids (e.g., blood plasma and plasma, urine, bronchial aspirate, pleural fluid, and exhaled breath condensate) [3]. Therefore, new insights into disease molecular etiology, diagnostic and efficacy markers, or potential therapeutic targets will probably arise from the study of tumor cell metabolism.

The metabolic patterns associated with lung cancer have been explored before by several groups, using either direct nuclear magnetic resonance (NMR) spectroscopy or other methods. Some results have been coincident in different published studies showing the presence/alteration of specific metabolites. However, there is clear variability, especially because of the lack of a full clinical characterization of patients or standardized patients’ selection [20].

Although there have been numerous studies on the use of metabolomics in lung cancer diagnosis, tumor characterization, and progression, little research has been conducted on the relationship between plasma metabolomic profiles and clinical outcomes in this population. Previous studies have attempted to address this gap in knowledge, such as the work conducted by Shen et al. [21]. The authors aimed to investigate the importance of blood metabolites in the prediction of OS among advanced-stage NSCLC patients who received platinum-based CTX. Four metabolites were identified, namely caffeine, paraxanthine, stachydrine, and methyl glucopyranoside (alpha + beta), which were significantly different between NSCLC patients with poor and good survival. These metabolites may serve as promising biomarker candidates to help identify patients who may benefit from platinum-based CTX. In a recent study by Tian et al. [22], pre-treatment plasma metabolic profiles from NSCLC patients treated with platinum-doublet CTX were analyzed. The authors identified a metabolite panel consisting of seven metabolites (hypotaurine, uridine, dodecanoylcarnitine, choline, dimethylglycine, niacinamide, and L-palmitoylcarnitine), which was significantly associated with longer median progression-free survival (PFS) (10.3 vs. 4.5 months, *p* < 0.001). Hao et al. proposed a novel hypothesis that the elevation of glutathione synthesis, supported by elevated methylation pathways, could be associated with improved survival in NSCLC patients who undergo CTX [23]. In their study, the authors observed that elevated levels of blood 2-hydroxybutyrate, glycine, and formate were all positively associated with better OS in NSCLC patients. Additionally, sphingolipids were found to be positively associated with OS. These findings suggest that measuring specific blood metabolites could provide valuable prognostic information for NSCLC patients undergoing CTX. The same group also published a study in which they evaluated the plasma metabolomic profiles of 25 lung cancer patients undergoing CTX with or without radiation, to assess the role of metabolites as biomarkers of temporal clinical outcomes [24]. The authors identified metabolites such as hydroxylamine, tridecan-1-ol, and octadecan-1-ol, which were indicative of better survival, while metabolites such as tagatose, hydroxylamine, glucopyranose, and threonine were reflective of cancer progression. Botticelli et al. conducted a study on the metabolomic profile of gut microbiota in 11 NSCLC patients who received nivolumab as a second-line treatment [25]. They found that 2-Pentanone (ketone) and tridecane (alkane) were significantly associated with early progression, while short-chain fatty acids such as propionate and butyrate, as well as lysine and nicotinic acid, were associated with significant long-term beneficial effects. These findings suggest that the gut microbiota may play a role in the response to IT and that metabolites produced by the microbiota could serve as potential biomarkers for predicting clinical outcomes in NSCLC patients. In the realm of IT and metabolomics, Nie et al. [26] sought to utilize early on-treatment plasma metabolomic profiling to identify predictors of clinical outcomes in advanced NSCLC patients undergoing anti-PD-1 treatment. They identified and validated a plasma metabolite panel consisting of hypoxanthine and histidine as a predictor of response to PD-1 blockade treatment. High levels of both metabolites in early on-treatment plasma were associated with improved PFS and OS.

In this work, we intended to study the metabolomic profile of patients with Adenocarcinoma (AdC), and their evolution during treatment, particularly after three and six treatments. Additionally, our study aimed to identify metabolic alterations and metabolic pathways responsible for the mechanisms that lead to a positive therapeutic response, enabling the identification of potential markers to guide clinical decision-making.

## 2. Materials and Methods

This work has been carried out in close collaboration between the University Hospitals of Coimbra (CHUC), the Faculty of Medicine (FMUC), and the Institute of Biomedical Sciences Abel Salazar—University of Porto (ICBAS-UP). The recruitment of patients occurred at the oncology unit of the pulmonology department of the CHUC. Patients over 18 years of age and with advanced lung AdC were included. This study was exploratory, prospective, and observational. The sequential recruitment was carried out over twenty-four months (from April 2018 until March 2020). Unfortunately, it had to be suspended due to the emergence of new rules for the handling of samples and biofluids, as well as restrictions on access to health care services in the context of the SARS-CoV-2 pandemic.

All selected patients were informed of the purpose of the study and the methodology that was going to be used. The privacy and confidentiality of the data of the participants were protected during all the process. All patients signed an informed consent for participation in research studies in accordance with the requirements of the Portuguese *Direção Geral da Saúde*, the FMUC ethics committee (process code CE 039/2018), and the Helsinki Declaration.

### 2.1. Subjects

In total, 18 lung cancer patients with AdC were included in this study. Six (33.3%) were female, and twelve (66.7%) were male. The mean age was 69.5 ± 10.6 years old, ranging from 44 to 75 years old. Final diagnosis and staging were established via histopathological evaluation, according to the 2015 World Health Organization (WHO) classification of lung tumors and the 8th TNM lung staging system. Based on the TNM staging system, tumors were classified as stage IIIA (n = 3, 16.7%), stage IIIB (n = 1, 5.5%), stage IIIC (n = 3, 16.7%), stage IVA (n = 7, 38.9%), and stage IVB (n = 4, 22.2%). All the patients underwent biomarker molecular testing, and no target fusion, mutation, or rearrangement was detected. All subjects included in this study were evaluated for comorbidities using the Charlson comorbidity index (CCI). It is one of the most frequently used index to measure co-existing pathologies. It is also used for predicting the risk of mortality, disability, hospitalization, and length of hospital stay in various clinical settings. CCI is age-dependent and evaluates several pathological conditions, including cardiovascular disease, chronic obstructive pulmonary disease (COPD), liver disease, and peptic disease, as well as diabetes, the presence of lymphoproliferative diseases, and solid tumors [27]. To characterize other features of the study population, several data were recorded, namely age, gender, smoking habits, profession, housing type, usual medication, family background, tumor type and stage, therapeutic regimens, and imaging evolution. Several biochemical parameters were also evaluated throughout the treatments (glycated hemoglobin, hemoglobin, leucocytes, platelets, urea nitrogen, creatinine, glucose, sodium and potassium levels, osmolality, triglycerides, and cholesterol), as well as patients’ weight and body mass index (BMI). To better characterize the study population, the authors decided to separately evaluate the population of long responders. These are the ones who remained under the therapeutic scheme for 12 or more months.

As mentioned above, 18 AdC patients were included in this study. Regarding smoking habits, 10 patients were non-smokers; 3 were smokers at the time of the inclusion in this study, with an average smoking burden of 83 ± 20 pack-years (minimum 60 and maximum 100 pack-years); and 5 patients were former smokers, with an average smoking burden of 52 ± 14 pack-years (minimum 32 and maximum 70 pack-years).

Most patients had other associated co-morbidities, namely arterial hypertension (n = 6), dyslipidemia (n = 8), diabetes mellitus (n = 1), cardiac pathology (n = 2), history of stroke (n = 1), and osteoarticular pathology (n = 3). Associated pulmonary pathology was also frequent, such as Chronic Obstructive Pulmonary Disease (COPD) (n = 6), sleep apnea (n = 1), and sarcoidosis (n = 2). One patient had a history of previous colorectal cancer. Three patients had no previous or associated co-morbidities. According to CCI, our population had a mean index of 2 ± 1.4 besides the AdC, which is associated with a 90% estimated 10-year survival. The incorporation of metastatic solid tumors in the index calculation confers a 0% estimated 10-year survival with a mean index value of 8. There were no significant differences between the demographic characteristics of the two study groups of patients (Table 1).

**Figure 1:**
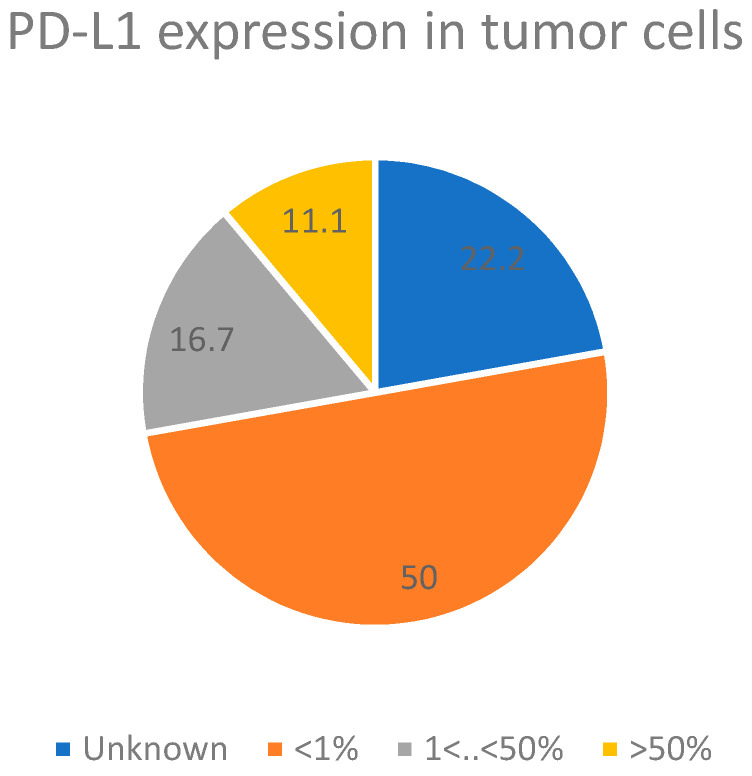
PD-L1 expression in tumor cells of the enrolled patients.

Additionally, we characterized the expression of PD-L1 in tumor cells of each enrolled patient (Figure 1).

**Figure 2:**
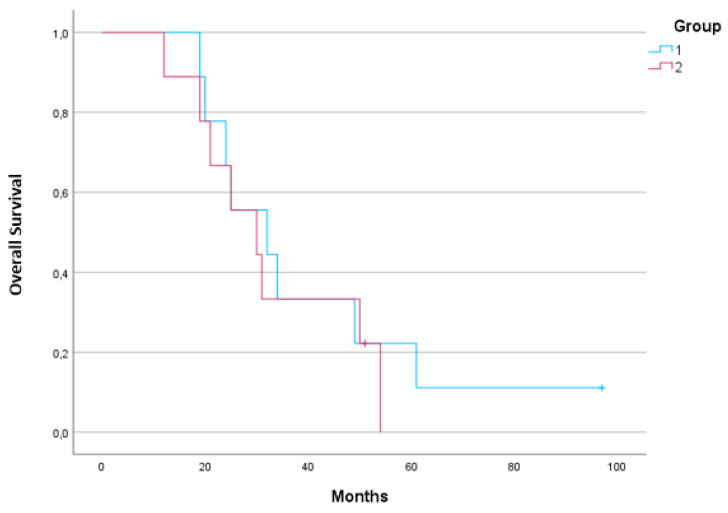
Kaplan–Meier curves comparing the cumulative survival between patients under immunotherapy (IT) vs. chemotherapy (CTX). (Blue line represents group 1—IT patients, N = 9; red line represents group 2—CTX patients, N = 9).

Complete information on the demographic and histopathological data of the patients enrolled in this study can be found in Table 2. Data concerning the biochemical characterization of the sera were collected throughout the period of follow-up, as well as patients’ weight and BMI. These values were recorded in the three moments of the collection of the samples (T0, T3, and T6) and can be found in Table 3. The response to the treatment regimen was performed using a computed tomography (CT) lung scan and was carried out at two moments in the follow-up with the patients: after the third (T3) and sixth (T6) treatments. These results are included in Table 4, according to RECIST 1.1 and iRECIST guidelines [28,29]. As mentioned above, this study included 9 patients under IT and 9 under CTX. These patients remained under the line of treatment used in this study for an average of 11.3 ± 5.9 months, with longer mean times in patients under IT vs. CTX (13.0 vs. 9.7 months). The differences between these treatments durations times are not statistically different in a significant way (*p*-value = 0.247). It was also verified that these patients had a mean OS after diagnosis of 37.4 months (95% CI: 27.021–47.837 months), being slightly higher in patients under IT (40.11; 95% CI: 24.418–55.805 months) vs. CTX (32.89; 95% CI: 22.4–43.376 months). The comparison between these two groups, using a Kaplan–Meier curve, showed that there is no statistically significant difference in the survival times (*p*-value = 0.617) in these patients under different therapeutic regimens (Figure 2).

### 2.2. Therapeutic Regimens

Therapeutic regimens were decided for each individual patient according to histological type, TNM staging, performance status (PS), co-morbidities, previous treatments, biomarkers, PD-L1 level of expression, and analytical results. All the therapeutic options were decided in a multidisciplinary meeting, according to the latest NCCN and ESMO guidelines, at the time of the beginning of the treatment. They were explained and discussed with the patient and family. Informed consent was obtained, as well as all the possible complications and secondary effects explained. Patients who completed CTX therapeutic regimens followed doublet platinum-based regimens (Cisplatin or Carboplatin plus Pemetrexed). The main therapeutic drugs that were prescribed are summarized in Table 5. The therapeutic regimen used in each patient is detailed in Table 6.

### 2.3. Sample Collection

During the follow-up of patients undergoing CTX or IT, imaging control is recommended in order to assess the effectiveness of the therapy. This evaluation is usually performed every 2 months. Based on this procedure, all patients provided blood prior to the beginning of the therapy (T0). Peripheral blood was also collected at the end of the third (T3) and sixth (T6) treatments, according to the therapeutic scheme that the patient had been prescribed. The packaging and transport of the samples was carried out in accordance with the standard rules and the processing of the biological samples took place at the Coimbra Institute for Clinical and Biomedical Research (iCBR). The principal investigator, as a member of the pulmonologist team of the oncology unit, was responsible for selecting patients as well as collecting the samples. In order to minimize dietary influence, all biofluid samples were collected in the morning, after overnight fasting, and no control over previous food intake was performed. Blood was collected through venipuncture and stored into sodium heparin tubes. To separate the plasma, avoid cell rupture/leakage, and minimize transport of metabolites between intra- and extracellular compartments, blood was centrifuged (1500× *g*, 10 min) for a maximum of 30 min. Plasma aliquots of approximately 1 mL were then transferred into sterile cryovials, frozen, and stored at −80 °C until analysis.

### 2.4. NMR Spectroscopy

The analyzed metabolites were assessed via proton nuclear magnetic resonance (^1^H-NMR) and spectra analysis. One-dimensional ^1^H-NMR spectra were acquired on a 600 MHz Bruker Avance spectrometer equipped with a 5 mm QXI probe with a z-gradient (Bruker Biospin, Karlshruhe, Germany) at 298 K. A zgpr pulse sequence for water suppression was used, and 32 scans were acquired for each sample. To obtain our results, 480 µL of plasma was mixed with cold methanol in a ratio (1:2 *v*/*v*) and incubated at −20 °C for 30 min. Then, samples were centrifuged at 13,000× *g* for another 30 min. The supernatant was then transferred to a new Eppendorf tube and subsequently dried in a rotary concentrator. Samples were resuspended in 600 µL of sodium fumarate solution in D_2_O ([fumarate] = 2 mM). Sodium fumarate was used as an internal reference for metabolite quantification in the media. The quantified metabolites were the following (multiplicity, chemical shift (ppm)): isoleucine (doublet, 0.99), valine (doublet, 1.02), alanine (doublet, 1.46), acetate (singlet, 1.90), lactate (quartet, 4.11), glucose (double, 5.22), tyrosine (multiplet, 6.89), and formate (singlet, 8.40). The spectra were manually phased and baseline corrected. NUTS-Pro^TM^ NMR software (Acorn NMR, Inc., Fremont, CA, USA) was used to integrate the chosen metabolite peaks. The concentration of the metabolites is expressed in mM.

### 2.5. Statistical Analysis

Descriptive results are expressed in mean ± standard deviation (SD) or n (%). Independent t-test was used to compare the means presented in the results section to determine if there was a significant difference between the two groups. In qualitative variables, associations were verified using the Chi-squared test, with Fisher’s correction when necessary. Kaplan–Meier curves were calculated to express OS. A *p*-value < 0.05 indicated statistical significance. Statistical analysis of the study group was performed using IBM SPSS 25.0 Statistics™. Univariate analysis of metabolites concentrations is represented as Tukey’s boxplot (median, 25th to 75th percentiles ± 1.5 IQR). In this case, statistical analysis was performed in GraphPad Prism 9 (GraphPad Software, San Diego, CA, USA). The Kolmogorov–Smirnov test was used to test data for normal distribution. Statistical differences were determined using a two-tailed unpaired *t*-test or one-way ANOVA, with post hoc Tuckey’s multiple comparisons test. *p*-value < 0.05 was considered significantly different. Multivariate statistical analysis was applied using MetaboAnalyst 5.0. Data on metabolite concentration were submitted through log transformation (base 10) before analysis. Then, principal component analysis (PCA) was performed to provide qualitative information on the observed data set.

## 3. Results

### 3.1. General Metabolomic Profile—Univariate Analysis

**Figure 3:**
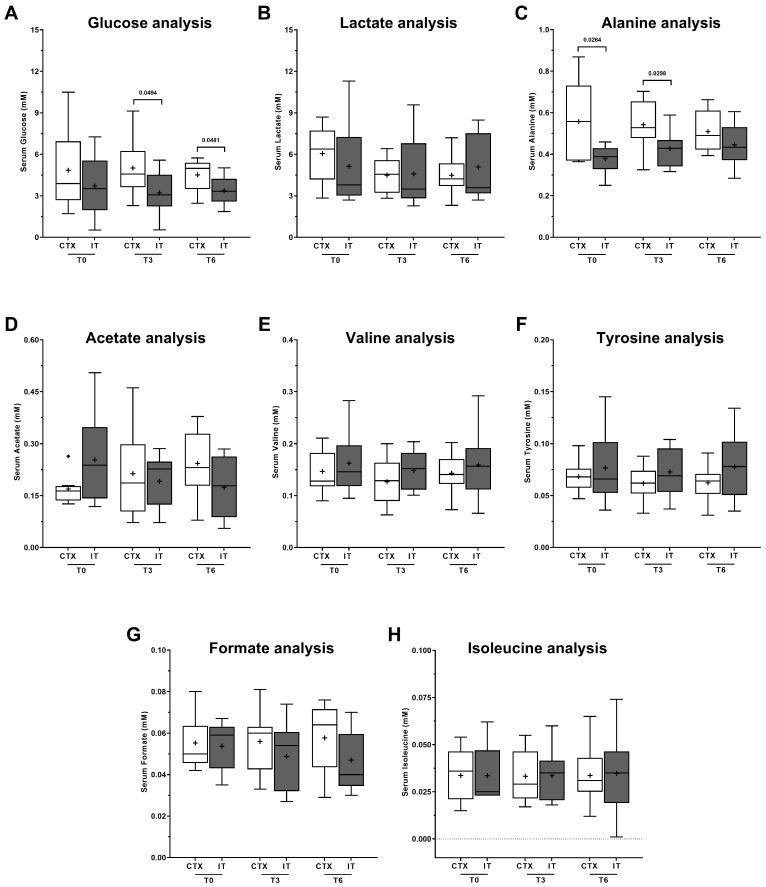
Metabolic profile of the plasma collected from patients submitted to chemotherapy (CTX) (N = 9) or immunotherapy (IT) (N = 9). Plasma samples were collected at three moments of the treatment: T0 (before), T3 (after 3 cycles), and T6 (after 6 cycles). Results are expressed as Tukey’s boxplot (median, 25th to 75th percentiles ± 1.5 IQR). + represents group average.

^1^H-NMR spectra were analyzed to assess the metabolic profile of the plasma upon CTX or IT treatments, as well as the three moments of the therapeutic process. There was a statistically significant change (*p*-value < 0.05) in the glucose plasma values, when we compared patients undergoing CTX and IT, at T3 (*p*-value = 0.049) and T6 (*p*-value = 0.048). Significantly higher values were found in patients undergoing CTX, when compared to IT, particularly with continued treatment (higher values at T3 and T6) (Figure 3A). Data also show that with the continuation of CTX, the plasma levels of glucose in these patients became higher. This trend was not observed in the IT patients. Another important result is that CTX-treated patients were characterized by significantly (*p*-value < 0.05) different levels of the amino acid alanine. In particular, patients who underwent CTX are characterized by higher plasma levels of the above-mentioned metabolite (Figure 3C) in moments T0 (*p*-value = 0.0264) and T3 (*p*-value = 0.0298). Furthermore, it has been confirmed that continuing the treatment leads to a decrease in the levels of alanine, bringing them closer to the levels observed in patients undergoing traditional IT. This suggests that patients who qualify for CTX may have higher levels of this specific amino acid in their system.

There was no statistically difference observed in the plasma levels of lactate, acetate, valine, tyrosine, formate, and isoleucine at the three moments of the treatment analyzed, and no difference was also found between CTX and IT therapeutic regimens (Figure 3B,D–H).

### 3.2. General Metabolomic Profile—Multivariate Analysis

With the present work, we aimed to evaluate the metabolomic profile of plasma from patients with AdC, in three moments: before starting treatment (T0) and after three (T3) and six (T6) treatments. Our goal was also to compare the metabolomic profile of patients undergoing CTX and IT to identify differences and patterns that can inform treatment selection and predict patient response. Through analyzing and comparing the metabolic profiles of these patients, we hope to gain insights into which treatment may be more effective for certain patient populations. Ultimately, our findings could help guide clinical decision-making and improve patient outcomes. In order to achieve these aims, we applied a multivariate analysis to non-targeted metabolomics data to compare the plasma metabolites from patients who underwent CTX and IT. The scatter plots we obtained revealed no significant influence of the treatment process on plasma metabolome composition, which can be observed in the lack of clustering trends.

**Figure 4:**
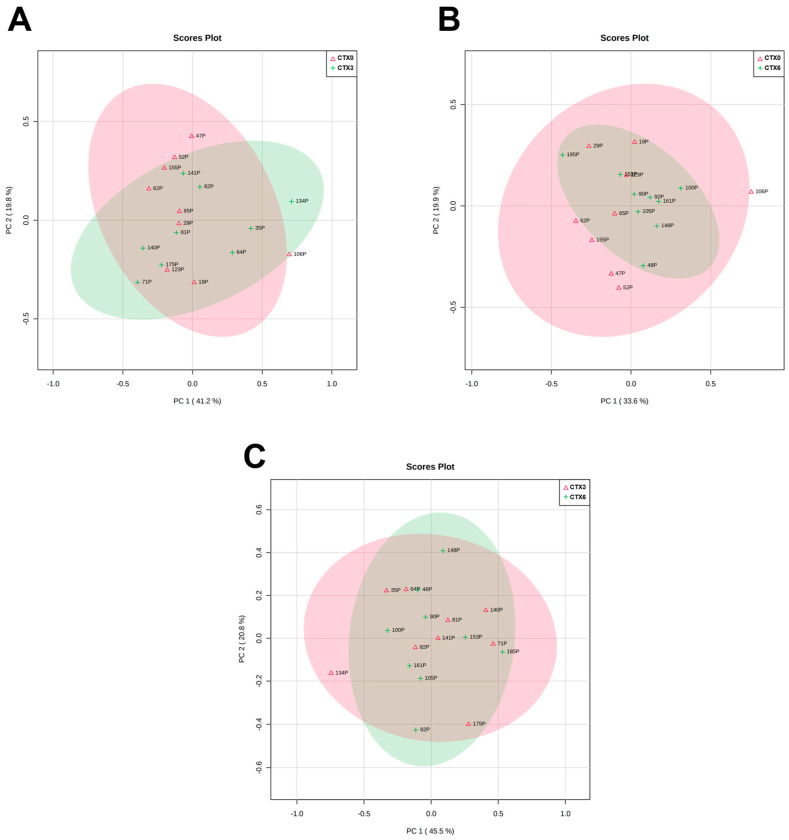
Scatter plots to chemotherapy treated patients (N = 9). (**A**)—Comparison between metabolomic findings in T0 (CTX0) and T3 (CTX3). (**B**)—Comparison between metabolomic findings in T0 (CTX0) and T6 (CTX6). (**C**)—Comparison between metabolomic findings in T3 (CTX3) and T6 (CTX6). Each number represents one plasma sample. The correspondence to patients’ identification can be seen in Table 6.

**Figure 5:**
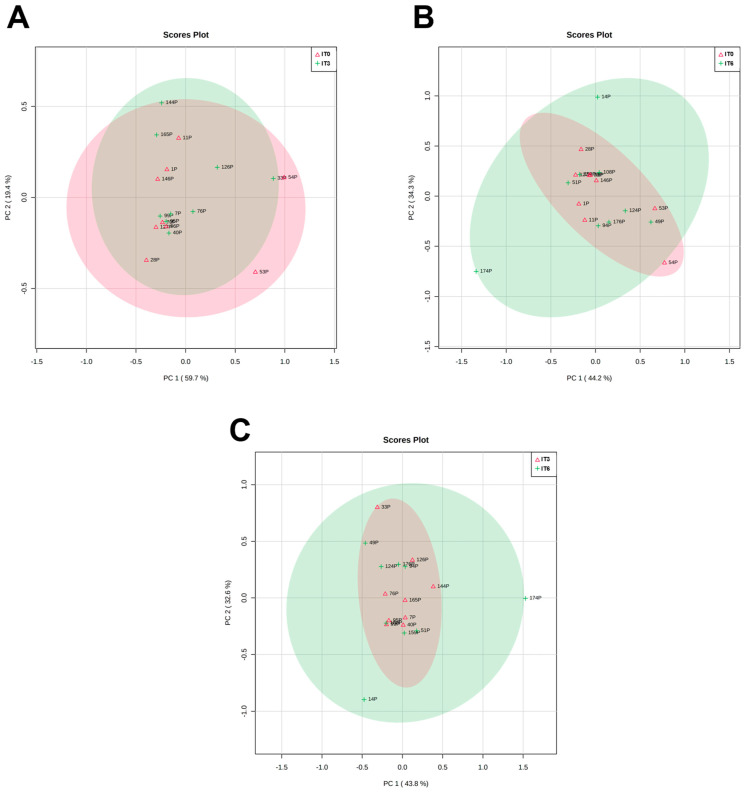
Scatter plots to immunotherapy treated patients (N = 9). (**A**)—Comparison between metabolomic findings in T0 (IT0) and T3 (IT3). (**B**)—Comparison between metabolomic finding in T0 (IT0) and T6 (IT6). (**C**)—Comparison between metabolomic findings in T3 (IT3) and T6 (IT6). Each number represents one plasma sample. The correspondence to patients’ identification can be seen in Table 6.

**Figure 6:**
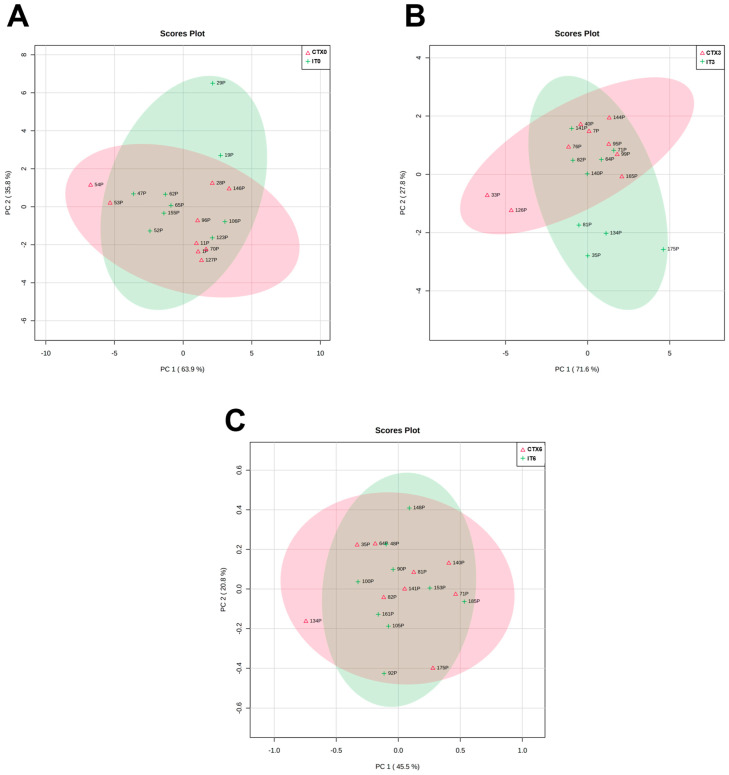
Scatter plots to patients included in the study. (**A**)—Comparison between metabolomic findings in T0, CTX (N = 9, CTX0) vs. IT (N = 9, IT0). (**B**)—Comparison between metabolomic findings in T3, CTX (CTX3) vs. IT (IT3). (**C**)—Comparison between metabolomic findings in T6, CTX (CTX6) vs. IT (IT6). Each number represents one plasma sample. The correspondence to patients’ identification can be seen in Table 6.

PCA was not able to identify a discrimination pattern in patients according to the different timings of the treatment, in any type of treatment (Figure 4 and Figure 5). Moreover, no significant alterations or patterns were detected comparing the three moments of treatment with CTX and IT, nor when comparing these two therapies in the same moments of the treatment (Figure 6). As no global pattern or tendency was identified, it was necessary to analyze each patient who appeared outside of the global trend. Figure 4 shows that samples belonging to patient number 14 deviate from the global characteristics of the other included patients. This deviation is also evident in Figure 5, where the patient’s results at T0 and T3 are different from those of other patients. The patient in question is a 56-year-old man with a smoking burden of 90 packs-years and a history of COPD and alcoholism and a family history of colon cancer. He was diagnosed with stage IV AdC and treated with carboplatin doublet and pemetrexed. Analytically, the patient exhibited a decrease in hemoglobin at T6 and blood glucose levels above 300 mg/dL at T6, along with high total cholesterol levels before the start of therapy. However, the patient’s disease was stable according to the RECIST criteria. In Figure 4, patient number 6 appears separate when comparing T0 with T6 and T3 with T6. This situation could suggest a change in the metabolomic profile induced by the therapy. Patient 6 is a 53-year-old non-smoking woman undergoing therapy with cisplatin and pemetrexed doublet. She has a history of sarcoidosis and a family history of prostate cancer. Notably, her recorded parameters include a BMI corresponding to obesity and a triglyceride level of 306 mg/dL. Despite this, the patient’s disease remained stable throughout the imaging evaluation.

In Figure 4, two patients are separated from the group. Patient 1 appears separate in the comparison between T0/T6 and T3/T6, while Patient 8 is separated in the comparison between T0/T3 and T0/T6. Both patients are men diagnosed with AdC in stage IIIA and have negative PD-L1 expression. They also have a history of dyslipidemia, arterial hypertension, and overweight.

Patient 1 is 69 years old, has COPD, and has a family history of gastric cancer. He is a smoker with a tobacco load of 60 pack-years. During treatment, he experienced an increase in triglyceride levels and a partial response in the imaging evaluation. Patient 8 is a former smoker with a load of 32 pack-years, and no analytical alterations were registered. However, he presented imaging progression of the disease at the end of the sixth treatment. Patient 2 is also outside the overall pattern in Figure 5, particularly in T3 and T6, indicating some alteration during the treatment. He is a 63-year-old man with a smoking history of 60 pack-years and a medical history of arterial hypertension, dyslipidemia, and COPD. Additionally, he has a family history of prostate cancer. Analyzing the analytical profile, he maintained stability in the registered parameters throughout the three evaluation moments. In the imagological evaluation, the disease remained stable in T3 and showed a partial response in T6, with a 30% reduction in the volume of the main tumor mass and a decrease in lymph nodes and liver metastases. Although these patients are outside the global pattern in the PCA results, there are no apparent distinguishing or common features among them.

### 3.3. Long Responders’ Metabolomic Profile—Univariate Analysis

To gain a better understanding of the clinical significance of the observed alterations in glucose and alanine levels, we focused our analysis on the long responders’ patients, defined as those who remained under the therapeutic scheme for 12 or more months. In the CTX group, we identified five patients with an average treatment duration of 13.2 (±1.3) months, while in the IT group, there were six patients with a significantly longer average treatment duration of 18 (±5.5) months (*p*-value = 0.046). This comparison allowed us to assess the potential impact of treatment duration on the observed metabolic changes in glucose and alanine levels.

**Figure 7:**
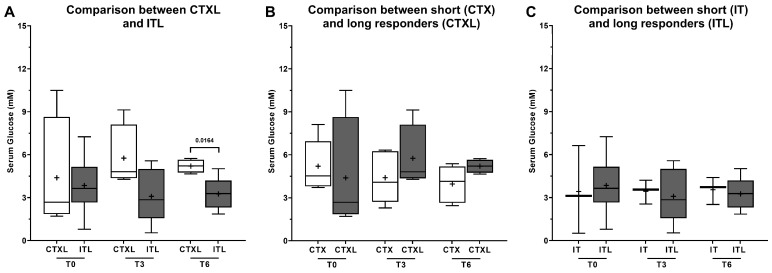
Glucose levels in plasma collected from patients submitted to chemotherapy (CTX) or immunotherapy (IT). Plasma samples were collected at three moments of the treatment: T0 (before), T3 (after 3 cycles), and T6 (after 6 cycles). Results are expressed as Tukey’s boxplot (median, 25th to 75th percentiles ± 1.5 IQR). +—Represents group average. (**A**) Comparison between long responders under CTXL (N = 5) and ITL (N = 6). (**B**) Comparison between long responders (CTXL) and the rest of the patients under CTX (N = 4). (**C**) Comparison between long responders (ITL) and the rest of the patients under IT (N = 3).

**Figure 8:**
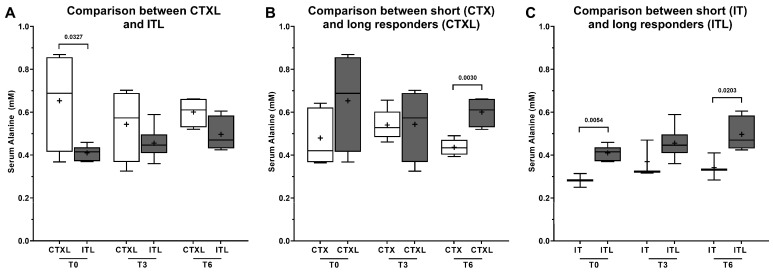
Alanine levels in plasma collected from patients submitted to chemotherapy (CTX) or immunotherapy (IT). Plasma samples were collected at three moments of the treatment: T0 (before), T3 (after 3 cycles), and T6 (after 6 cycles). Results are expressed as Tukey’s boxplot (median, 25th to 75th percentiles ± 1.5 IQR). +—Represents group average. (**A**) Comparison between long responders under CTXL (N = 5) and ITL (N = 6). (**B**) Comparison between long responders (CTXL) and the rest of the patients under CTX (N = 4). (**C**) Comparison between long responders (ITL) and the rest of the patients under IT (N = 3).

**Figure 9:**
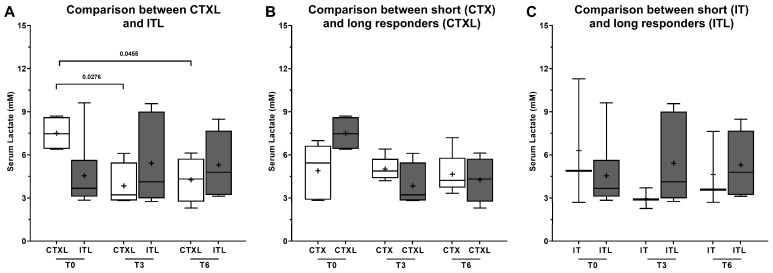
Lactate levels in plasma collected from patients submitted to chemotherapy (CTX) or immunotherapy (IT). Plasma samples were collected at three moments of the treatment: T0 (before), T3 (after 3 cycles), and T6 (after 6 cycles). Results are expressed as Tukey’s boxplot (median, 25th to 75th percentiles ± 1.5 IQR). +—Represents group average. (**A**) Comparison between long responders under CTXL (N = 5) and ITL (N = 6). (**B**) Comparison between long responders (CTXL) and the rest of the patients under CTX (N = 4). (**C**) Comparison between long responders (ITL) and the rest of the patients under IT (N = 3).

One notable finding in the NMR spectra was a statistically significant increase (*p*-value = 0.016) in plasma glucose levels at T6 in long responders receiving CTX, compared to those undergoing IT (Figure 7A). This increase in plasma glucose levels with continued CTX treatment was not observed in patients undergoing IT. Furthermore, long responders receiving CTX were characterized by significantly (*p*-value = 0.0327) higher levels of the amino acid alanine before the beginning of the treatment (Figure 8A), supporting the hypothesis that CTX-eligible patients have higher levels of alanine. Comparing the long responders receiving CTX to the rest of the CTX population (Figure 8B, *p*-value = 0.0030), and the same for the IT population (Figure 8C, *p*-value = 0.0203), they showed significantly higher levels of alanine in T6, suggesting that long responders maintain higher levels of this amino acid throughout the treatment. The levels of lactate showed a significant difference in the long responders’ group, but not in the total sample analysis. Specifically, in patients treated with CTX, the levels of plasma lactate were higher at T0 compared to T3 (*p*-value = 0.0276) and T6 (*p*-value = 0.0455) (Figure 9A). However, no other statistically significant differences were observed in the levels of acetate, valine, tyrosine, formate, and isoleucine among long responders’ patients, regardless of the therapeutic scheme used and the three moments of treatment analyzed.

### 3.4. Long Responders’ Metabolomic Profile—Multivariate Analysis

**Figure 10:**
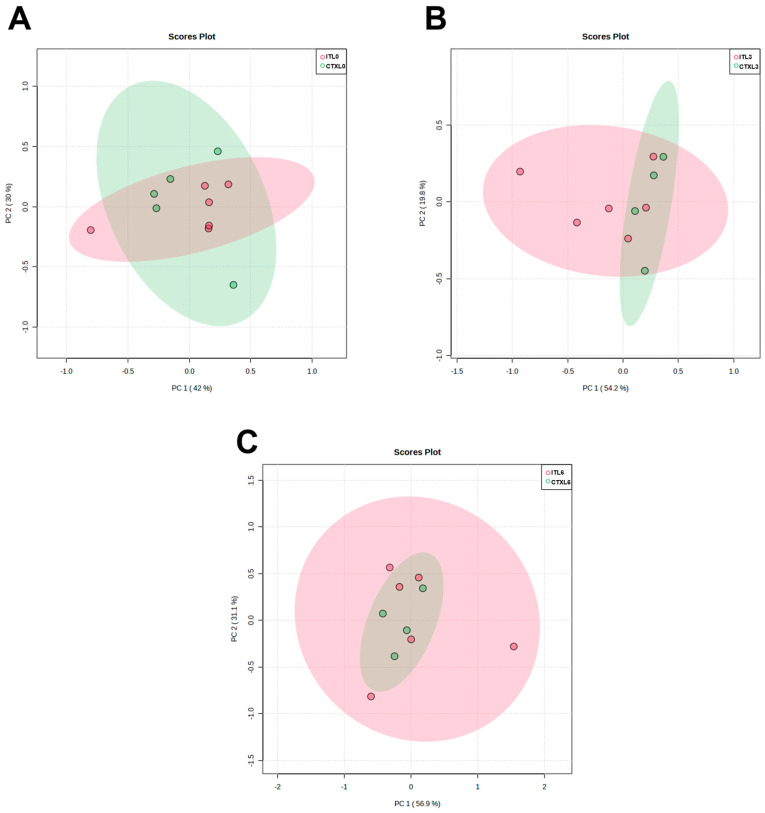
Scatter plots including long responders’ patients. (**A**)—Comparison between metabolomic findings in T0, CTX (QT0) (N = 5) vs. IT (IT0) (N = 6). (**B**)—Comparison between metabolomic findings in T3, CTX (QT3) vs. IT (IT3). (**C**)—Comparison between metabolomic findings in T6, CTX (QT6) vs. IT (IT6). Each dot represents one plasma sample.

To compare the plasma metabolome composition of long responders’ patients, we conducted a PCA. In line with the results from the total sample analysis, the scatter plots showed no significant clustering trends that could be attributed to the treatment process. Specifically, we did not identify any discrimination pattern or metabolite that could distinguish long responders’ patients at different times of treatment or under different therapeutic regimens (Figure 10). These findings suggest that the observed metabolic changes in glucose and alanine levels are not indicative of broader shifts in the global plasma metabolome composition.

## 4. Discussion

Based on literature review, it is evident that treatment has a significant impact on the metabolomic evolution of lung cancer patients. Our findings suggest that glucose plasma values could be significantly affected by treatment, with higher values observed in patients undergoing CTX compared to those undergoing IT, particularly with continued treatment (higher values at T3 and T6). When comparing long responders undergoing CTX and IT, higher glucose plasma values were also found in patients undergoing CTX, particularly at T6. These results are consistent with previous studies, such as the one conducted by Wikoff et al., which demonstrated a significant reduction in glucose levels in AdC tissue compared to non-malignant tissue, suggesting an increased pentose phosphate metabolism and elevated glucuronidation status in AdC [30]. Moreno et al. also reported a significant decrease in various glycolysis metabolites involved in AdC and Squamous cell carcinoma (SqCC), such as glucose, 3- and 2-phosphoglycerate, and phosphoenolpyruvate, accompanied by a significant accumulation of lactate and pyruvate, the final products of glycolysis [31]. To further understand the potential impact of corticosteroid premedication and adjuvant medication on the observed differences in glucose levels between patients undergoing CTX and IT, additional studies are needed. While the study by Bordag et al. [32] suggests that corticosteroid treatment can have a significant impact on the metabolome, it is unclear whether the dose and duration of corticosteroid treatment in lung cancer patients undergoing CTX in our study is sufficient to explain the observed differences. Future studies could consider including a control group of patients receiving corticosteroid treatment without CTX to better understand the specific impact of corticosteroid treatment on glucose levels in lung cancer patients. Additionally, studies could explore other potential factors, such as differences in diet or physical activity between patients undergoing CTX and IT, which could contribute to the observed differences in glucose levels. Our analysis of HbA1c values showed no significant statistical difference (*p*-value = 0.9409, CI 95%: −0.6033 to 0.647) between patients undergoing CTX and IT. The same result was obtained when we compared glucose plasma levels from patients under CTX vs. IT (*p*-value = 0.6523, CI 95%: −94.676 to 60.964). Moreover, we did not find any significant differences between the two subgroups in terms of their history of diabetes mellitus. Therefore, we could not identify any other explanation for the differences in glucose levels other than the confounding factor of premedication and adjuvant medication and a possible response to therapy.

Moreno et al. [31] reported a significant decrease in the levels of various glycolysis metabolites and a significant accumulation of lactate in tumor subtypes, AdC and SqCC. In our study, we also found a significant difference in the levels of lactate in the long responders’ group, which was not observed in the total sample analysis. Interestingly, in the CTX-treated patients, the levels of plasma lactate were higher at T0 compared to T3 and T6, which is consistent with the lower levels of glucose detected at the beginning of the treatment. However, as the treatment progressed, we observed a significant increase in glucose levels and a decrease in lactate levels, further supporting a possible response to therapy. The “Warburg effect” is a well-known phenomenon in cancer cells, characterized by an increase in glycolytic activity and a decrease in glucose levels, leading to elevated lactate levels. This phenomenon has been reported in lung tumors as well, as evidenced by increased lactate and decreased glucose levels [33,34]. In fact, Rocha et al. [20] found that this variation was more pronounced in SqCC than in AdC, where glucose and lactate levels were significantly negatively correlated. Recent studies have also shown that lactate plays an important role in the tumor microenvironment and can affect various immune cells. Lactate can suppress the function of immune cells such as T cells and natural killer (NK) cells, which are important in the antitumor immune response [35]. Additionally, lactate can promote the differentiation of immunosuppressive cells like myeloid-derived suppressor cells (MDSCs) and regulatory T cells (Tregs) [36]. These cells can inhibit the immune response and promote tumor growth. Therefore, lactate not only affects tumor cells but also the immune cells in the tumor microenvironment, which can have significant implications for tumor growth and response to therapy. Numerous studies have demonstrated that intratumoral lactate levels are markedly elevated, reaching up to 40 mM, whereas normal tissues exhibit levels of 1.8–2 mM. This metabolic shift towards high lactate levels has been strongly associated with cancer resistance and poor outcomes [37]. Our findings are consistent with these studies, as we observed a decrease in lactate levels in the long responders group with continued therapy, which is indicative of a favorable response and prognosis.

Our study also revealed significantly different levels of the amino acid alanine in CTX-treated patients (*p*-value < 0.05). Specifically, these patients had higher plasma levels of alanine at T0 and T3 compared to patients under IT. Interestingly, with the continuation of the treatment, the levels of alanine decreased and became more similar to those observed in patients under IT. These findings suggest that CTX treatment may impact alanine metabolism, and monitoring alanine levels could be a potential biomarker for assessing response to therapy. Further research is needed to elucidate the underlying mechanisms and clinical implications of these observations. Several studies have investigated the role of amino acids in lung cancer metabolism, with few focusing on the potential role of alanine. One study by Klupczynska et al. [38] identified a set of six amino acids (aspartic acid, β-alanine, histidine, asparagine, phenylalanine, and serine) that improved sample classification accuracy, while Maeda et al. [39] identified a model that could discriminate between NSCLC patients and controls based on the concentrations of alanine, valine, isoleucine, histidine, tryptophan, and ornithine. However, there has been limited research on the relationship between alanine levels and chemotherapeutic response in lung cancer. In our study, we observed higher levels of alanine in CTX-treated long responders before the start of treatment. Furthermore, when comparing long responders under CTX and the rest of the CTX-treated population, as well as those under IT, significantly higher levels of alanine were detected at T6, indicating that long responders maintained higher levels of this amino acid. These findings suggest that plasma levels of alanine may serve as a potential biomarker for predicting durable responses to CTX. Recently, Ghini et al. [14] used metabolomics to analyze sera samples from 50 NSCLC patients treated with IT. Their study demonstrated that the metabolomic fingerprint of plasma could act as a predictive “collective” biomarker for ICI response, accurately predicting individual therapy outcomes with over 80% accuracy. In particular, the study found that non-responder and responder subjects treated with nivolumab exhibited significantly different levels of the amino acid alanine and pyruvate, with responders showing lower levels of these metabolites. Interestingly, similar findings were observed in pembrolizumab-treated patients, suggesting that metabolomic prediction of ICI response may be independent of the specific ICI agent used. While these results may appear to contradict the findings of our study, further research is needed to draw definitive conclusions. Regarding alanine, it is worth noting that low levels of this amino acid have been shown to serve as a marker for sarcopenia and frailty, both in healthy populations and in patients with chronic illness. For instance, low plasma alanine activity was found to be associated with shortened survival in middle-aged healthy adults [40] as well as in patients hospitalized for various causes [41]. Furthermore, low alanine values, which are associated with low muscle mass and sarcopenia, may represent increased frailty and subsequently shortened survival. Dagan et al. investigated the hypothesis that low levels of alanine could act as a predictive marker of poor prognosis in lung cancer patients. However, in this paper, the results showed that low levels of alanine were not associated with an increased risk of mortality [42]. This hypothesis could provide a plausible explanation for the reduction in alanine levels observed throughout the course of therapy in our patients, given the fragility and adverse reactions induced by CTX. As patients undergo CTX cycles, they may become more debilitated, leading to immobilization and a potential loss of muscle mass, which could contribute to the development of sarcopenia. Moreover, since all patients prescribed with IT had previously completed a CTX scheme, they may have already been in a state of greater fragility, which could explain the lower levels of alanine before the beginning of the IT scheme. The association between lower levels of alanine and sarcopenia is further supported by the finding that long responders maintained higher levels of this amino acid. Nevertheless, larger, prospective studies are necessary to validate this hypothesis.

It is also important to acknowledge the limitations of this study. It is essential to highlight that this study was exploratory, containing a small sample size. The small size of the sample, as well as the inclusion of stage III and IV patients, and the lack of a control group (healthy individuals) do not allow definitive conclusions to be drawn about the OS, as well as about the metabolites’ variations. Studies with larger cohorts are needed to validate the obtained results. Additionally, the applied metabolomics technique has some limitations. Although it is characterized by its high reproducibility and sample recovery, ^1^H-NMR has limited sensitivity and can only quantify a small number of metabolites without further processing of the samples. Therefore, the multivariate analysis was performed with limited data on metabolites. In this study, the multivariate PCA did not reveal any distinctive features or differentiation patterns in the analysis of all included patients and the long responders group. The lack of a clear pattern could suggest that both the differences between groups and timepoints occur in specific metabolites and pathways, making it difficult to obtain a clear separation between groups, and the differences found in only two or three metabolites may not be enough to achieve separation in PCA. Therefore, in our study, the univariate analysis is particularly important. During the development of our study, there were many difficulties and challenges: mainly the emergence of the COVID pandemic, which limited the inclusion of patients, as well as the storage and handling of samples. These restrictions did not allow us to increase the sample size, leading to a decrease in the significance of the obtained results. Future studies using alternative methods, including mass spectrometry or multi-omics approaches, should be conducted to validate and complement our results.

## 5. Conclusions

In conclusion, our analysis of plasma samples from our cohort of AdC patients revealed that only three metabolites—glucose, lactate, and alanine—were significantly altered. While our initial aim was to identify metabolomic patterns that could aid in predicting individual outcomes of CTX and IT, we did not observe any clear pattern in our sample. However, our findings may open the path to study the suggestion that higher glucose levels and lower lactate levels during the course of CTX could be associated with tumor response, while lower levels of alanine could indicate greater frailty, disease progression, and poor prognosis. Our study highlights and encourages the future use of NMR metabolomics for improving the diagnosis, treatment, and prognosis of AdC, and we hope to confirm and expand upon our findings in a larger study, including patients with different histopathological features.

## Figures and Tables

**Table 1:** Comparison of baseline characteristics from the two study groups.

	CTX Group N = 9	IT Group N = 9	*p*-Value
Age (years)	61.56 ± 9.50	60.67 ± 8.44	0.840
Smoking habits (pack-years)	41.11 ± 41.67	14.67 ± 23.15	0.116
Gender—Female (%)	17.20	44.40	0.317
CCI	2 (0–5)	3 (0–3)	0.446
Treatment duration (months)	9.78 ± 4.24	13 ± 7.19	0.247

**Table 2:** Demographic and histological information on lung cancer patients enrolled in this study.

Patient Number	Diagnosis	Staging	PD-L1 Level	Age	Sex	Smoking Habits	Profession	Housing Type	Co-Morbidities (CCI)	Family History of Cancer
1	AdC	IIIA T4N0M0	Neg	69	M	S—60 SPY	Retired (legal assistant)	Rural house	HT, COPD, Dysl (3)	Stomach
2	AdC	IVA T4N1M1a	10%	63	M	FS—40 SPY	Retired (budgetist)	Rural house	HT, Dysl, OSA, AMI (3)	Prostate
3	AdC	IVA T4N0M1a	?	69	F	NS	Farmer	Rural house	0 (2)	Lung
4	AdC	IVA T4N0M1a	Neg	57	F	NS	Stay-at-home mom	Rural house	0 (1)	Breast
5	AdC	IVA T4N3M1a	Neg	75	M	FS—70 SPY	Retired (policeman)	Rural house	COPD, DM, HT, Dysl (5)	Larynx
6	AdC	IIIC T4N3M0	Neg	53	F	NS	Stay-at-home mom	Rural house	Sarcoidosis (1)	Prostate
7	AdC	IIIC T3N3M0	90%	62	M	S—100 SPY	Construction	Rural house	Dysl (2)	0
8	AdC	IIIA T2bN2M0	Neg	65	M	FS—32 SPY	Electrician	City apartment	HT, Dysl (2)	0
9	AdC	IVA T3N3M1b	70%	47	M	NS	?	City apartment	0 (0)	0
10	AdC	IIIA T2bN2M0	?	44	M	NS	Scrap worker	City apartment	0 (0)	0
11	AdC	IVB T3N0M1c	Neg	65	M	NS	Construction	Rural house	AF, HT, Dysl (2)	0
12	AdC	IVA T4N0M1a	?	69	F	NS	Stay-at-home mom	Rural house	0 (5)	Lung
13	AdC	IVA T3N2M1b	20%	52	F	NS	Stay-at-home mom	Rural house	Dysl (2)	0
14	AdC	IVA T4N0M1a	Neg	56	M	S—90 SPY	Cleaning open spaces	Rural house	COPD, alcoholism (2)	Colon
15	AdC	IIIB T3N2M0	?	61	M	FS—70 SPY	Retired (construction)	City apartment	Dysl, HT, myocardiopathy (3)	0
16	AdC	IIIC T4N3M0	Neg	54	F	NS	Stay-at-home mom	Rural house	Sarcoidosis, Amaurosis (1)	Prostate
17	AdC	IVB T3N3M1c	10%	69	M	FS—50 SPY	Retired (bank officer)	City apartment	Colon tumor (2)	Larynx
18	AdC	IVA T1N1M1b	Neg	70	M	NS	Retired (truck driver)	Rural house	HT (19	Colon

AdC—adenocarcinoma; CCI—Charlston Comorbidity Index; M—male; F—female; S—smoker; FS—former smoker; NS—non-smoker; SPY—smoking pack-years; HT—arterial hypertension; COPD—chronic obstructive pulmonary disease; Dysl—dyslipidemia; AF—atrial fibrillation; DM—diabetes mellitus; OSA—obstructive sleep apnea; AMI—acute myocardial infarction.

**Table 3:** Biochemical data of patients enrolled in this study (T0/T3/T6).

Patient No	Weight (Kg)	BMI (Kg/m^2^)	Hemoglobin (g/dL)	Glyc Hb (%)	Leucocytes (×10^9^/L)	Platelets (×10^9^/L)	Sodium (mmol/L)	Potassium (mmol/L)	Urea Nitrogen (mg/dL)	Creatinine (mg/dL)	Glucose (mg/dL)	Osmolarity (mOSM/Kg)	Triglycerides (mg/dL)	Cholesterol (mg/dL)
1	71/71/71	26.7/26.7/26.7	14.1/14.9/14.8	5.8	5.9/6.4/7.4	213/227/226	132/135/134	4.8/4.4/4.4	11/12/13	0.98/1.02/1.19	99/99/97	264/271/268	181/170/281	203/214/200
2	63/67/71	21.7/23.2/24.7	12.4/13.3/14	6	8.5/7.1/9.3	266/296/278	140/139/138	3.9/4.3/4.3	16/13/15	0.74/0.73/0.72	111/104/115	280/278/277	118	118
3	73/72/73	27.9/27.5/27.6	13.8/13.2/11.3	6.5	12.3/8/7.6	223/260/195	138/138/139	4/3.6/3.2	19/24/23	0.66/0.66/0.69	276/203/244	286/286/289	247	224
4	52/52/51	22/22/21.5	13.2/13.4/13	6.6	12.7/10.8/12.7	299/279/371	140/139/136	4.7/3.8/3.7	15/16/13	0.72/0.7/0.62	127/116/122	281/270/273	111	309
5	63/65/65	21.8/22.5/22.5	14.1/10.7/10.5	6.6	6.6/3.7/3.7	205/364/307	137/136/135	3.9/4.8/5.1	14/26/20	0.79/0.93/0.95	277/289/284	284/287/283	73	133
6	99/101/97	31.9/32.6/31.3	11.3/11.1/11	5.4	8.9/6.5/8.9	249/417/373	132/130/132	4.6/5.1/5.4	11/8/12	0.73/0.67/0.72	128/112/114	266/260/265	304	93
7	60/62/64	20.3/20.1/21.6	13.8/13.5/12.1	6.2	15/6.2/6.6	200/346/404	139/140/139	4.1/3.9/3.9	15/14/14	0.6/0.74/0.69	93/149/154	278/283/276	62	115
8	84/84/77	26.8/26.8/24.3	12.2/11.2/10.1	6.6	6.1/7.7/7.8	206/288/347	139/141/137	5.1/4.6/4	13/23/17	0.86/0.87/0.75	87/72/116	272/284/276	193	32
9	74/70/71	22.4/21.1/21.4	9.7/13.3/13.8	5.7	13.7/5.3/5.7	754/343/344	137/138/140	4.2/4.5/3.9	9/16/23	0.74/0.77/0.84	131/87/89	274/274/283	176	182
10	75/68/65	24.6/22.2/21.2	14/13.4/12.2	5.8	11.5/3.5/3.6	250/289/222	137/138/138	4/4.2/4.4	16/10/10	0.87/0.77/0.93	113/113/115	275/276/278	51	185
11	68/67/69	27.3/26.9/27.6	16/15.2/15	5.8	7.0/5.1/6.9	279/252/214	141/142/141	3.9/3.8/3.9	10/19/17	0.66/0.82/1	115/94/95	282/285/283	115	180
12	75/71/69	28.6/27.1/26.1	14/13.5/13.1	7.5	6.9/6.9/6.2	170/196/105	143/138/141	3.8/4.1/3.9	17/17/19	0.65/0.61/0.89	128/139/92	286/285/283	247	224
13	42/44/44	20/21/21	9.1/10.9/11	5.3	8.8/3.6/4.9	583/381/351	136/136/139	4.2/4,1/4.4	10/13/18	0.75/0.74/0.69	198/107/131	277/278/281	80	183
14	50/47/49	16.9/16.3/16.6	12.8/11.7/8.5	5.5	7.3/4.1/5.5	186/316/395	138/142/139	4.8/5.1/5.1	8/6/9	0.81/0.73/0.83	141/154/305	276/284/288	69	259
15	79/73/73	24.9/22.9/22.9	16/12.8/12.7	6.8	9.4/5.9/7.5	338/419/415	140/140/140	5.1/5.3/4.7	33/22/25	1.17/1.32/1.36	196/165/233	272/286/293	95	163
16	99/95/95	37.7/36.2/36.2	11.2/10.9/11.2	5.4	5.2/4.2/5.4	213/241/233	134/132/131	3.9/4.7/4.5	8/8/10	0.91/0.68/0.83	103/85/86	267/262/261	304	310
17	107/104/105	32.3/31.4/32	14.3/11/11.1	5.9	10.7/5.4/8.9	277/333/397	134/135/136	4.5/5/4.9	15/11/10	0.78/0.78/0.74	169/141/158	273/272/274	59	189
18	113/101/99	35.2/33.4/31	8.8/10/10.3	5.4	6.4/5.6/6.9	223/213/243	142/140/140	4/3.6/3.4	11/12/11	0.75/0.74/0.64	102/144/144	283/282/281	49	219

BMI—Body Mass Index; Hb—Hemoglobin.

**Table 4:** Imaging evaluation of patients enrolled in this study, performed via CT lung scan after the third (T3) and sixth (T6) treatments, according to RECIST 1.1 and iRECIST criteria.

Patient No	T3	RECIST 1.1 iRECIST	T6	RECIST 1.1 iRECIST
1	Reduction of main tumor nodule (26 × 23→19 × 16 mm)	PR	Reduction of main tumor nodule, stable size of lymph nodes	PR
2	Heterogeneous response—reduction and increase of different nodules	SD	Reduction of 30% of the volume of the main lesion, reduction of lymph nodes and liver metastasis	PR
3	Stability	SD	Stability	SD
4	Suspected pseudoprogression	PP	Emergence of pleural effusion	PD
5	Small reduction of main tumor (47 × 28→40 × 25 mm)	SD	Increase of the main tumor, carcinomatous lymphangitis	PD
6	Small reduction of main tumor nodule	SD	Stability	SD
7	Small reduction of main tumor	SD	Reduction of main tumor (38 × 26→26 × 19 mm)	PR
8	Small reduction of main tumor	SD	Increase of the main tumor (36 × 44→52 × 59 mm)	PD
9	Small reduction of main tumor	SD	Reduction of main tumor (54 × 74 × 54→30 mm)	PR
10	Stability	SD	Increase in number and size of lymph nodes, carcinomatous lymphangitis	PD
11	Stability	SD	Stability	SD
12	Stability	SD	Small reduction of main tumor	SD
13	Suspected metastatic involvement of D11	PD	Stability	SD
14	Stability	SD	Stability	SD
15	Stability	SD	Stability of main tumor, reduction in number and size of lymph nodes	SD
16	Stability	SD	Increase of main tumor and lymph nodes	PD
17	Stability of main tumor, reduction of lymph nodes	SD	Reduction of main tumor (48 × 26→29 × 16 mm)	PR
18	Stability	SD	Reduction of main tumor and number and size of lymph nodes	PR

RECIST—Response Evaluation Criteria in Solid Tumors; PR—partial response; SD—stable disease; CR—complete response; PD—progressive disease; PP—pseudoprogression.

**Table 5:** Main therapeutic drugs used in the patients enrolled in this study.

Drug	Dose
Chemotherapy	
Cisplatin	75 mg/m^2^, day 1, every 21 days
Carboplatin	AUC 5 or 6, day 1, every 21 days
Pemetrexed	500 mg/m^2^, day 1, every 21 days
Immunotherapy	
Nivolumab	240 mg, every 2 weeks
Pembrolizumab	200 mg, every 3 weeks
Atezolizumab	1200 mg, every 3 weeks

**Table 6:** Therapeutic scheme used in the enrolled patients and identification of the patients’ code used in the metabolomic analysis.

Patient	Therapeutic Scheme	T0 Sample Code	T3 Sample Code	T6 Sample Code
1	Nivolumab	1P	7P	14P
2	Pembrolizumab	11P	33P	49P
3	Carboplatin + Pemetrexed	19P	35P	48P
4	Nivolumab	28P	40P	51P
5	Cisplatin + Pemetrexed	29P	71P	92P
6	Cisplatin + Pemetrexed	47P	64P	90P
7	Cisplatin + Pemetrexed	52P	81P	100P
8	Nivolumab	54P	95P	108P
9	Pembrolizumab	53P	76P	95P
10	Carboplatin + Pemetrexed	62P	82P	105P
11	Carboplatin + Pemetrexed	65P	141P	153P
12	Nivolumab	70P	99P	124P
13	Pembrolizumab	96P	126P	159P
14	Carboplatin + Pemetrexed	106P	134P	148P
15	Carboplatin + Pemetrexed	123P	140P	161P
16	Atezolizumab	146P	165P	174P
17	Carboplatin + Pemetrexed	155P	175P	185P
18	Nivolumab	127P	144P	176P

## Data Availability

All data of this article are available in the Figures and Tables. Raw data are made available on request to the corresponding author. Data are not publicly available due to privacy.
